# Differences in Parental Burnout: Influence of Demographic Factors and Personality of Parents and Children

**DOI:** 10.3389/fpsyg.2018.00887

**Published:** 2018-07-02

**Authors:** Sarah Le Vigouroux, Céline Scola

**Affiliations:** ^1^Social Psychology Laboratory, University of Nîmes, Nîmes, France; ^2^Center for Research on the Psychology of Cognition, Language and Emotion, Aix-Marseille University, Marseille, France

**Keywords:** parental burnout, personality, parents, children, dispositional factors

## Abstract

Parental burnout is a syndrome related to parenthood and characterized by three dimensions: emotional and physical exhaustion, emotional distancing of parents from their children, and loss of parental accomplishment. Many factors can explain the interindividual differences in parental burnout (Roskam et al., 2017). In a study conducted among 372 French parents, we examined the relationship between parental burnout, demographic factors (age of parent and child(ren), age of parent at first birth, total number of children, and number of children present in the family home) and parent-assessed dispositional factors (personality traits of parent and child(ren)). Results for demographic factors showed that the younger the parents we surveyed, the higher their reported sense of parental accomplishment, although they also tended to feel more exhausted. We observed a similar pattern of results when we looked at the children’s ages. In addition, the number of children at home slightly increased the emotional distance between parent and child(ren). Results for the parents’ dispositional factors showed that all three personality traits we investigated, as well as their different facets (lack of emotional control and lack of impulse control for *neuroticism*, meticulousness and perseverance for *conscientiousness*, and cooperation and friendliness for *agreeableness*), were related to parental burnout and its three dimensions. More specifically, parental meticulousness and lack of emotional control were both risk factors for developing parental burnout. By contrast, agreeableness and perseverance were protective factors. With regard to the children, the same three personality traits were linked to the three dimensions of parental burnout. Having children they perceived as having a high level of neuroticism reduced parents’ sense of parental accomplishment and increased their emotional exhaustion and distancing. The opposite relationships were found for agreeableness and conscientiousness. There were no significant relationships between parental assessments of their children’ extraversion and openness and parental burnout and its three dimensions. The parent’s personality explained 42.3% of the variance in parental burnout, and the child(ren)’s personality (parent-assessed) explained 13.8%. Taken together, these results demonstrate the importance of considering the personality of both parents and children in the study of parental burnout.

## Introduction

Parental burnout is a syndrome consisting of three dimensions (Roskam et al., 2017): emotional and physical exhaustion (the parent feels completely drained of energy); emotional distancing from the child(ren) (the parent no longer has the strength to invest in the emotional relationship he/she has with his/her child(ren) and has only functional interactions with them); and a sense of inefficacy in his/her parenting role (the parent no longer derives any feeling of parental accomplishment from his/her parenting role).

Recent research has attributed the triggering of this syndrome to an accumulation of demographic, situational and dispositional risk factors (Lindström et al., 2011; Mikolajczak et al., 2017; Roskam et al., 2017). Possible risk factors for parental burnout have been explored in populations of parents of either healthy (Mikolajczak et al., 2017) or sick (with Type 1 diabetes; Lindström et al., 2011) children. Neither of these studies found a link with any sociodemographic factor [e.g., parent’s sex and age, child(ren)’s sex and age, number of children, parent’s education and marital status], apart from having young children aged below 5 years, which was a risk factor. Conversely, the parent’s situational and dispositional factors appeared to play an important role. The situational factor highlighted in both these studies was a dysfunctional family circle, and more particularly dissatisfaction with conjugal relationships. With regard to parental disposition, low self-esteem and a high need for control (Lindström et al., 2011), as well as low emotional intelligence and an avoidance of attachment (Mikolajczak et al., 2017), were identified as risk factors for parental burnout.

Parental personality also seems to contribute to the risk of developing parental burnout (Le Vigouroux et al., 2017; Mikolajczak et al., 2017). Individuals with very high levels of neuroticism (i.e., emotionally unstable) are at greater risk of developing depressive syndrome (DeNeve and Cooper, 1998; Thompson et al., 2015) and have been found to exhibit stricter parenting behavior (Prinzie et al., 2009). Le Vigouroux et al. (2017) found that a high level of neuroticism was also a risk factor for burnout in the parents of typical children, as was a low level of conscientiousness or agreeableness. However, this study was based on a brief 10-item measure of personality (Ten-Item Personality Inventory; Ehrhart et al., 2009) that did not take the specific facets of personality traits into account. These facets offer a more fine-grained and accurate measure of individual behavior, and may have differential effects on parental burnout. For example, Fayard et al. (2012) highlighted differences between the facets of conscientiousness (e.g., industriousness vs. traditionality) in terms of the experience of guilt. Moreover, parental personality (especially emotional stability, conscientiousness, and agreeableness) influences the development of children’s personality. This influence mainly takes place through childrearing practices, and alters the quality of the parent–child relationship (Denissen et al., 2009; Schofield et al., 2012).

*Emotional instability* (i.e., neuroticism) corresponds to frequent worries, as well as to more frequent and more intense negative affect (McNiel and Fleeson, 2006; Finch et al., 2012). In particular, this trait encompasses emotional control and impulse control behaviors. Emotionally unstable parents respond more intensely to life events (Prinzie et al., 2009). This tendency to exhibit negative emotionality impairs childcare, as well as the ability to initiate and maintain positive emotional interactions with children, and limits parents’ ability and willingness to respond adequately to their children’s signals. Neuroticism is of primary importance in determining parental burnout, followed by conscientiousness and agreeableness. The importance of neuroticism is not surprising. Many studies have highlighted the role of this personality trait in the study of emotional states (e.g., Larsen and Ketelaar, 1991; McNiel and Fleeson, 2006; Steel et al., 2008; Finch et al., 2012).

*Conscientiousness* refers to self-discipline, order, and planning. In particular, this trait includes meticulousness and perseverance. Individuals with higher levels of conscientiousness report fewer negative affects, especially guilt, and are better able to automatically down-regulate negative affect (Fayard et al., 2012; Javaras et al., 2012). Conscientious parents adhere to educational standards, allowing children to have a more coherent and structured environment (Prinzie et al., 2009). Conscientious children are organized and more easily accept delays before rewards, facilitating positive parenting (Schofield et al., 2012).

*Agreeableness* is characterized by friendly and empathetic behaviors. In particular, it encompasses friendliness and cooperation. Agreeable individuals are helpful, warm, altruistic, generous, and loving (McCrae and Costa, 1991; Marsh et al., 2013). The most agreeable parents form more positive attributions for their children’s behavior. They are better able to identify and respond to their children’s needs (Prinzie et al., 2009). In addition, children’s agreeableness fosters warm relationships with their parents (Denissen et al., 2009).

A previous study (Le Vigouroux et al., 2017) suggested that parents who have difficulty initiating and maintaining positive emotional interactions with their children (high neuroticism), identifying and responding to their children’s needs (low agreeableness) or providing a coherent and structured environment (low conscientiousness) for their children are more likely to experience parental burnout syndrome. However, the results of this study need to be confirmed with a more precise and complex measurement of personality traits.

The parent–child relationship is both dynamic and complex, and to our knowledge, none of the studies published so far have investigated the influence of children’s dispositional factors on the occurrence of parental burnout. If parents’ personality plays an important role in burnout, in terms of risk and protective factors, then children’s personality may also play an important role. Parents’ characteristics influence both parenting practices and children’s characteristics, which together influence the well-being of both parents and children, as well as the quality of the relationship between them (Denissen et al., 2009; Prinzie et al., 2009; Schofield et al., 2012).

The present study had three main objectives. The first was to replicate and probe data reported in several previous studies. More specifically, we wished to validate findings regarding the weak or absent relationship between sociodemographic factors (age of parents and children, number of children) and parental burnout, and the importance of personality traits (neuroticism, conscientiousness, and agreeableness). We also wanted to go further, by looking at possible differences in relationships between the three dimensions of parental burnout and the demographic and dispositional variables we collected. Some relationships may not appear at the general level of parental burnout, but emerge at the specific level of the emotional exhaustion, emotional distancing, and loss of parental accomplishment dimensions. For example, with respect to demographic factors, we assumed that parents with many children would report a higher level of emotional distancing, as they would have to respond to more material and functional demands, and would have less time available for emotional interactions with their children.

The second objective of our study was to clarify the relationships between personality and parental burnout, based on a more precise and complex measure of personality that looked at individual facets. For example, the study by Lindström et al. (2011) found that parents with a high versus low need for control (facet of conscientiousness) were at greater risk of developing parental burnout syndrome. Facets of parental conscientiousness may therefore have a differential influence. Parents who are meticulous, but lack perseverance, may be at greater risk of exhaustion, while parents who persevere but are less meticulous may be less likely to be exhausted. Concerning the various facets of agreeableness, we hypothesized that cooperation has a greater impact than friendliness on parental burnout and its dimensions (more specifically, emotional distancing and emotional exhaustion). Finally, regarding the facets of emotional stability, we hypothesized that emotional control is more important than impulse control when it comes to parental burnout and its dimensions.

The third objective of our study was to explore the relationships between children’s personality traits and parental burnout. We hypothesized that children’s personality is either a risk or a protective factor for parental burnout syndrome. We predicted that the patterns of results would be identical to those found for parental personality, with relations between parental burnout and three personality traits: (1) neuroticism as a risk factor, as emotionally unstable children require more resources from the parent to regulate them; (2) conscientiousness as a protective factor, as conscientious children respect family rules more; and (3) agreeableness as a protective factor, as agreeable children adapt themselves more to their parents’ emotions.

## Materials and Methods

### Participants

The sample consisted of 372 parents (314 mothers) of non-clinical children. Their ages ranged from 23 to 65 years (*M =* 36.76, *SD* = 7.57), and they had had their first child between the ages of 19 and 47 (*M* = 29.66, *SD* = 4.44). The parents interviewed in this study had between one and five children (*M* = 1.81), and was aged between 0 and 35 years. Participants were recruited from social networks and came from various regions of France. The study met local ethical rules on non-invasive protocols involving healthy participants and did not require formal ethics committee approval. All participants signed an informed consent form, which outlined the conditions for taking part as well as for withdrawing from the study, if desired.

### Procedure

The study described here was part of a longitudinal data collection project extending over a 13-month period that was designed to study the timecourse of parental burnout and its links to other emotional variables. Only data recorded at the first measurement point were used in the present study. Participants responded to an online questionnaire, distributed over social networks, entitled *Emotions and Parenting*. Parents could only participate in the study if they had at least one child still living at home.

### Measures

Parental burnout was probed with the Parental Burnout Inventory (PBI: Roskam et al., 2017). This scale contains 22 items (α = 0.81) assessing emotional exhaustion (8 items; α = 93), emotional distancing (8 items; α = 0.76), and parental accomplishment or efficacy (6 items; α = 0.73). All items are rated on the same seven-point Likert scale: 0 (*Never*), 1 (A *few times a year or less*), 2 (*Once a month or less*), 3 (*A few times a month*), 4 (*Once a week*), 5 *(A few times a week*), and 6 *(Every day*). Total and factor scores are obtained by summing the appropriate item scores, with higher scores indicating greater burnout (parental accomplishment items are therefore reverse-scored).

Parents’ personality was assessed with the Alter Ego (French adaptation of the BFQ; Caorara et al., 1992), a questionnaire that uses the Big Five dimensions to measure personality traits. In view of previous results, we chose to probe only three personality traits, each made up of two facets: emotional control (α = 0.84) and impulse control (α = 0.85) for *emotional stability* (the opposite of neuroticism; α = 0.89); perseverance (α = 0.72) and meticulousness (α = 0.80) for *conscientiousness* (α = 0.80); and friendliness (α = 0.66) and cooperation (α = 0.63) for *agreeableness* (α = 0.77). Each facet is probed with 12 items. All items are rated on the same five-point Likert scale ranging from 1 (*Quite wrong*) to 5 (*Quite true*). Factor scores are obtained by averaging the appropriate item scores.

The personality of children over 3 years^1^ was other-assessed (by the parent) with the French translation of the Ten-Item Personality Inventory (Gosling et al., 2003), which measures each of the five personality dimensions via two (opposite) items, rated on a seven-point scale ranging from 1 *(Doesn’t describe her/him at all*) to 7 (*Describes her/him very well*). Despite its brevity, this questionnaire has good convergent and predictive validity (Ehrhart et al., 2009), although in the present study, Pearson’s rank correlation coefficients for neuroticism, extraversion, conscientiousness, agreeableness, and openness were 0.37, 0.32, 0.31, 0.34, and 0.19, which is less than expected. Factor scores are obtained by averaging the appropriate item scores.

## Results

### Sociodemographic Factors

First, we tested the relationships between parental burnout (and its three dimensions) and the sociodemographic variables of our sample. Overall, correlations between demographic factors and parental burnout (**Table 1**) suggested that the number of children (regardless of whether they lived at home) and wide age gaps between these children were moderate risk factors for parental burnout syndrome. Neither the parent’s age at the time of study and at the birth of the first child nor the mean age of the children were correlated with parental burnout.

**Table 1:** Correlations between demographic factors and parental burnout.

	Parental burnout	Emotional exhaustion	Emotional distancing	Loss of parental accomplishment
Parental burnout	-			
Emotional exhaustion	0.85***	-		
Emotional distancing	0.73***	0.43***	-	
Loss of parental accomplishment	0.60***	0.21***	0.29***	-
Age of parent	0.01	-0.16**	0.05	0.24***
Age of parent at first birth	-0.03	-0.02	-0.11*	-0.05
Total number of children	0.15**	0.03	0.22***	0.14**
Number of children at home	0.12*	0.10*	0.16**	0.00
Mean age of children	0.04	-0.19***	0.10*	0.32***
Standard deviation for children’s age	0.13*	0.04	0.14**	0.15**


*^∗^*p* < 0.05; ^∗∗^*p* < 0.01; ^∗∗∗^*p* < 0.001.*

When we looked at the three dimensions of parental burnout (emotional exhaustion, emotional distancing, and loss of parental accomplishment), the correlations were much stronger and more numerous. Our results showed that the level of emotional exhaustion was higher among young parents and parents with young children than among older parents and parents with older children. Emotional distancing was greater among parents with many children in total (more important than the number of children at home) or children with large age gaps, than among parents with few children in total or children with small age gaps. This second dimension of parental burnout was also related, albeit weakly, to the age of the children and the age of the parent at the birth of the first child. The older the children and the younger the parent when his or her first child was born, the more likely that parent was to experience significant emotional distancing. Parental loss of parental accomplishment was greater among older parents and among parents with many children or children who were older than among younger parents and parents with few children or children who were still young.

### Parental Personality

Second, we looked at the relationship between parental dispositional factors and the three dimensions of parental burnout. A recent study (Le Vigouroux et al., 2017) had highlighted the influence of three personality traits on parental burnout (neuroticism, conscientiousness, and agreeableness), and in the present study, we wanted to confirm these results and take them further by looking at specific facets of personality.

**FIGURE 1:**
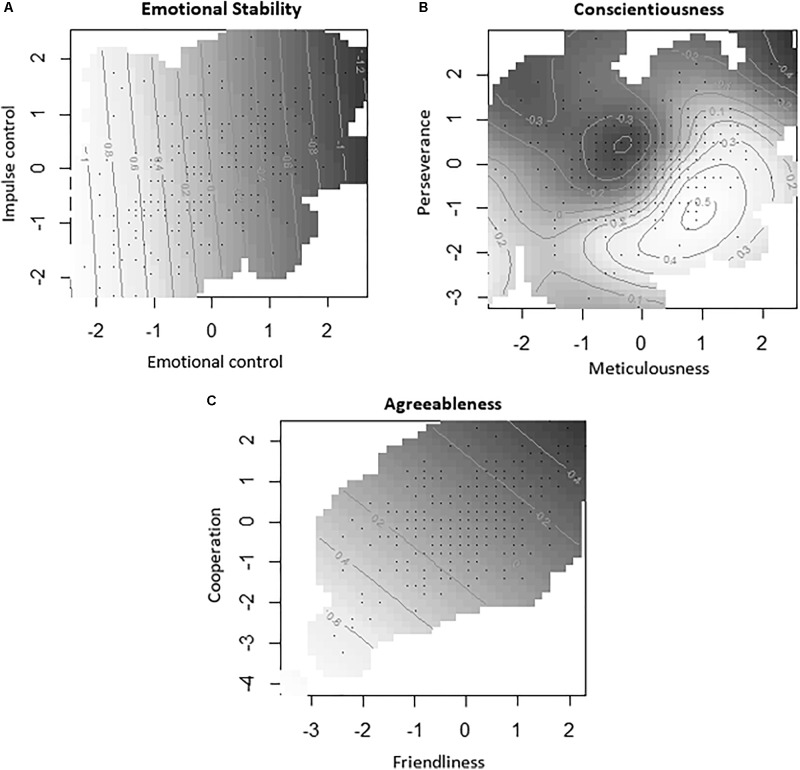
Estimation of parental burnout according to the personality facets of emotional stability **(A)**, conscientiousness **(B)**, and agreeableness **(C)**, based on generalized additive models (GAMs). The darker the zone, the lower the level of burnout.

Our results (**Figure 1** and **Table 2**), obtained by analyzing correlations and calculating generalized additive models (GAMs; McKeown and Sneddon, 2014), showed that agreeableness and emotional stability (i.e., opposite of neuroticism) protected against parental burnout. Emotional stability was most closely related to parental burnout and its three dimensions. Moreover we found that it was the control of emotions, more than the control of impulses, that protected against parental burnout. Parental conscientiousness was not related to parental burnout, but its two facets were. Thus, the more persistent parents were, the less parental burnout they experienced. By contrast, the more meticulous they were, the more parental burnout they experienced. To account for the strength of these relationships, we calculated GAMs that simultaneously considered the two facets of each personality trait and their impact on parental burnout (**Figure 1**). The proportion of deviance explained by each of the three models was 22.1% for emotional stability, 12.4% for conscientiousness, and 4.51% for agreeableness. The results are set out in **Figure 1** (the lighter the zone, the higher the estimated level of parental burnout per model, and the darker the zone, the lower the estimated level of parental burnout). A GAM that simultaneously considered the influence of emotional control, impulse control, perseverance, meticulousness, friendliness, and cooperation on parental burnout explained 42.3% of the variability of the data in our sample.

**Table 2:** Correlations between parental burnout (and its three dimensions) and parental personality traits (and facets).

	Parental burnout	Emotional exhaustion	Emotional distancing	Loss of parental accomplishment
Agreeableness	-0.20***	-0.21***	-0.12*	-0.10
Friendliness	-0.18**	-0.19***	-0.10	-0.08
Cooperation	-0.19***	-0.19**	-0.12*	-0.10
Conscientiousness	0.02	0.07	0.04	-0.12*
Meticulousness	0.12*	0.17**	0.09	-0.04
Perseverance	-0.12*	-0.06	-0.06	-0.17**
Emotional stability	-0.42***	-0.39***	-0.28***	-0.23***
Emotional control	-0.46***	-0.42***	-0.33***	-0.24***
Impulse control	-0.28***	-0.27***	-0.18**	-0.16**


*^∗^*p* < 0.05; ^∗∗^*p* < 0.01; ^∗∗∗^*p* < 0.001.*

When we looked at the specific dimensions of parental burnout (**Figure 1** and **Table 2**), we found that emotional exhaustion was the variable most influenced by the parent’s agreeableness, emotional stability, and meticulousness. Emotional distance was related to non-cooperative behaviors and emotional instability (high level of neuroticism). Loss of parental accomplishment was related to perseverance and emotional instability.

### Children’s Personality

Our third objective was to explore the links between children’s personality and parental burnout syndrome (and its three dimensions). When parents had two or more children, a separate burnout analysis was conducted for each child’s data. The results are set out in **Table 3**.

**Table 3:** Correlations between parental burnout (and its three dimensions) and children’s personality traits as assessed by parents.

	Parental burnout	Emotional exhaustion	Emotional distancing	Loss of parental accomplishment
Agreeableness	-0.26***	-0.18**	-0.21***	-0.19***
Conscientiousness	-0.18**	-0.13*	-0.14*	-0.12*
Extraversion	0.07	0.08	0.02	0.04
Neuroticism	0.24***	0.23***	0.15**	0.18**
Openness	-0.01	-0.03	0.01	-0.12**


*^∗^*p* < 0.05; ^∗∗^*p* < 0.01; ^∗∗∗^*p* < 0.001.*

Regarding the relationship between children’s personality and parental burnout, we observed the influence of the same three personality traits as we had done for parents: the more children were perceived of as being emotionally unstable, disagreeable, and non-conscientious, the higher the reported levels of parental burnout (no particular differences between the syndrome’s three dimensions). In addition, the parents of open children reported a higher sense of parental accomplishment. A generalized additive mixed model (GAMM) that simultaneously took children’s neurotic, conscientious and agreeable characteristics (as rated by the parent) into account explained 13.8% of the variability of the data in our sample.

**FIGURE 2:**
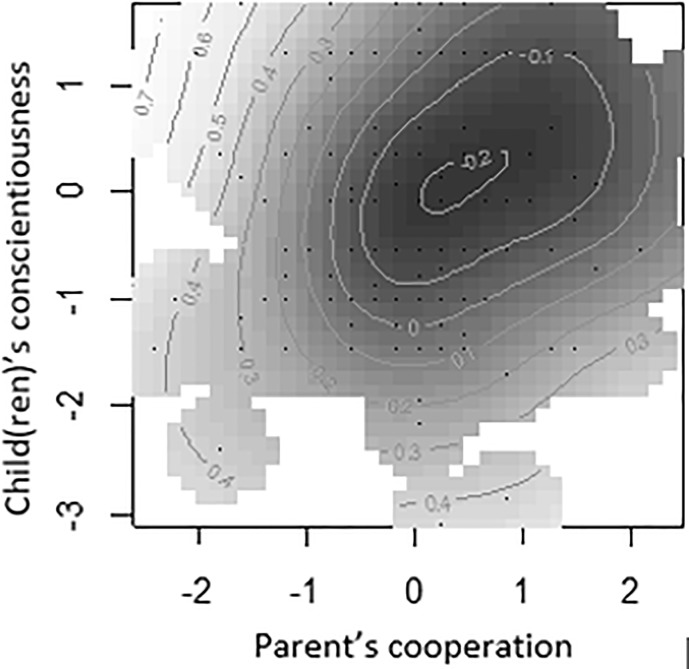
Effect of interaction between parental cooperation and child(ren)’s conscientiousness on parental burnout. The darker the zone, the lower the level of burnout.

We also wanted to explore the interactions between facets of the parent’s and child(ren)’s personalities, to study possible differences in their influence on parental burnout (e.g., what is the parental burnout level if the parent is agreeable but not the child, or if the parent is agreeable and the child is emotionally unstable?). Our results pointed to a cumulative effect of dispositional factors, with no interactions for most of the 18 configurations we tested, based on previous results (six personality facets for the parent and three personality traits for the child). The only interaction that emerged concerned the combined influence of the cooperative nature of the parent and the conscientiousness of the child on parental burnout (**Figure 2**), which explained 9.12% of the variance. Cooperative parents who perceived their child(ren) as conscientious had the lowest level of parental burnout, compared with uncooperative parents or parents who did not perceive their child(ren) as conscientious.

## Discussion

The present study was the first, to our knowledge, to examine the influence of both sociodemographic factors (age of parent, age of parent at first birth, total number of children, number of children at home, mean and standard deviation for children’s age) and dispositional factors (personality of parent and child(ren)) on parental burnout and its three dimensions. With regard to the dispositional factors, it seemed important to take into account not only the personality of the parent, but also that of the child(ren). Parental burnout syndrome manifests itself in the interactions between the parent and his or her children, and as this is a dynamic relationship, it seemed obvious to us that there was a reciprocal influence. It was therefore essential to take account of the children’s demographic and dispositional characteristics of the children. Our results confirmed the usefulness of this approach, as parental personality explained 42.3% of the variance of parental burnout, and child(ren)’s personality (parent-assessed) 13.8%. With regard to the dimensions of parental burnout, we felt it was important to study the relationships specific to each dimension (emotional exhaustion, emotional distancing, and loss of parental accomplishment), for while it is not necessary to have high scores on these three dimensions to exhibit parental burnout (Mikolajczak and Roskam, 2017; Mikolajczak et al., 2017), if some dimensions are strongly affected by certain factors, there may be a risk of developing parental burnout later on.

With regard to our first hypothesis concerning the influence of sociodemographic factors, our results confirmed the usefulness of studying parental burnout more precisely, looking at each specific dimension. When we looked at the general level of parental burnout, only three of the six sociodemographic variables we studied (total number of children, number of children living at home, and standard deviation for children’s age) were positively correlated and then only slightly with parental burnout. However, when these sociodemographic factors were analyzed in relation to each of the three dimensions of parental burnout, the number and strength of correlations increased.

Thus, (1) being a young parent was a risk factor for loss of parental accomplishment and emotional exhaustion, (2) being a first-time parent with a young child was a risk factor for emotional distancing from that child, (3) having many children was a risk factor for both emotional distancing and loss of parental accomplishment, (4) having many children at home was a risk factor for emotional distancing, (5) having infants was a risk factor for emotional exhaustion, while having adolescent children was a risk factor for both emotional distance and loss of parental accomplishment, and finally (6) a large age gap between the children was a risk factor for both emotional distancing and loss of parental accomplishment.

Taken together, these relationships show that the number of children, the age gap between these children, and certain developmental periods pose a greater risk in terms of parents’ emotional experiences. These results are consistent with our first hypothesis and can be explained in terms of parental practices and the parent’s personal development. Having one or more young children requires much more investment on the part of the parent than having children who are grown up and are becoming increasingly independent in their daily lives, just as having a large family multiplies the demands made on parents by their children. Moreover, adolescence is often a difficult time for parents, and from a societal perspective it also generates less satisfaction for them in their role. These results confirm the usefulness of exploring the different dimensions of parental burnout in the study of sociodemographic risk factors, as factors exerting a significant influence on one or two of these dimensions could potentially be combined with other factors to significantly reduce the prevalence of parental burnout.

With regard to our second hypothesis concerning the influence of parental dispositional factors, our results confirmed the relevance of considering the parent’s personality in relation to parental burnout and its three dimensions. More specifically, as expected, emotional stability (opposite of neuroticism) was the personality trait that afforded the greatest protection against parental burnout syndrome (Le Vigouroux et al., 2017), and acted more on the dimension of emotional exhaustion than on either emotional distancing or loss of parental accomplishment. More specifically, the emotional control facet influenced parental burnout more strongly than the impulse control facet. This result can be explained by the nature of parental burnout, which is situated on an emotional continuum between anxiety and depression (Mikolajczak and Roskam, 2017; Roskam et al., 2017). Agreeableness (including friendliness and cooperation) also proved to be a protective factor against parental burnout, and more particularly emotional exhaustion, but neither this trait nor its facets influenced the loss of parental accomplishment. The more detailed analysis of the facets of this trait revealed that cooperation protected against emotional distancing. This is an understandable relationship, as adopting cooperative behaviors toward others, especially children, helps to maintain positive emotional relationships (McCrae and Costa, 1991). The influence of conscientiousness on parental burnout syndrome also varied according to which facet we considered. Although the trait itself was not correlated with parental burnout, its meticulousness and perseverance facets were. Perseverance protected against parental burnout, and more specifically promoted a sense of parental accomplishment. Conversely, meticulousness was a risk factor for parental burnout, and more specifically increased the risk of emotional exhaustion. This result is consistent with the literature, which has shown that perfectionism (close to meticulousness) is a risk factor for parental burnout, as parents exhaust themselves by wanting to do too well (Lindström et al., 2011). This difference in conscientiousness may not have been picked up in Le Vigouroux et al. (2017)’s study, owing to the very brief tool used to assess the parents’ personality. The present study confirmed that parental personality is a key factor in the study of burnout, explaining almost half the variance (42%). Understanding the influence of personality traits on parental burnout syndrome is relevant, as these results can inform the design of psychological interventions. For example, mindfulness could be a useful means of working on emotional control or changing beliefs and behavior patterns.

With respect to our third hypothesis on the influence of child-specific dispositional factors, our results showed that the same three (parent-assessed) personality traits (neuroticism, agreeableness, and conscientiousness) were linked to parental burnout and its three dimensions. The more children were perceived of as being emotionally unstable, disagreeable and non-conscientious, the more parents reported high levels of parental burnout (with no particular differences across the syndrome’s three dimensions). This result confirmed our hypothesis that these three personality traits influence the emotional experiences of individuals interacting within the family. However, even though children’s personality was a major risk or protective factor for parental burnout, it influenced this syndrome less than the parent’s personality, which explained more than 40% of the variance.

The influence of dispositional factors (parent’s and child(ren)’s personality) on parental burnout is important to understand. Personality corresponds to the tendency of individuals to act, think and feel emotions. Understanding the behaviors, cognitions, and emotions of parents and children that are associated with parental burnout allows us to better understand this syndrome’s risk factors and thus to tailor psychotherapeutic interventions aimed at parents. Our results show that parents who do not control their emotions or who are very meticulous, report more burnout than parents who do or who are less meticulous. It would therefore be interesting to combine therapies promoting emotional skills with cognitive therapies that seek to modify the dysfunctional thoughts of exhausted parents.

## Conclusion

This article highlights the influence of sociodemographic and dispositional factors on parental burnout and its three dimensions. Our results emphasize the value of studying individual differences in parental burnout according to personality facets, in order to understand the risk and protective factors. This will not only help to shield parents from this syndrome, but also improve the management of burnout by professionals. There are several avenues worth investigating in future studies. First, it would be interesting to be able to explore children’s dispositional factors in greater detail. Because of the longitudinal nature of our project (here, only responses at the first measurement point were studied), we chose to use a brief measure of personality for the children. However, tools have been specifically adapted to children and adolescents, although they are longer and more costly experimentally. Similarly, this study did not explore the potential influence of very young children’s personality traits on parental burnout syndrome, although we were able to discuss the fact that having young children can be a risk factor for the manifestation of burnout, especially the emotional exhaustion dimension. Second, it would be interesting to take account of situational factors such as coparenting, marital satisfaction and social support, which may interact with dispositional factors. Third and last, a longitudinal study would determine whether parents scoring high on one or two burnout dimensions are more likely to go on to develop parental burnout.

## Author Contributions

CS co-supervised the data collection and co-authored the manuscript. SLV co-supervised the data collection, conducted the statistical analyses, and co-authored the manuscript.

## Conflict of Interest Statement

The authors declare that the research was conducted in the absence of any commercial or financial relationships that could be construed as a potential conflict of interest.

## Footnotes

1 For children under 3 years, we considered that it was not very valid to study personality according to the Big Five model of personality traits.
